# Around the Hospitals

**Published:** 1893-05-06

**Authors:** 


### Around the Hospitals.

Notice to the Managers of Institutions.—Special space is now reserved for the insertion of notices of hospital meetings and festivals. The Editor requests that all notices may reach The Hospital Office, 428, Strand, at least one week before the date of each meeting.

**The Bristol General Hospital.**—The experience of this hospital is hardly likely to form an encouraging example to those institutions which have decided to extend their work trusting to increased public generosity for maintenance. Since the new wing has been added to the Bristol General Hospital, far from a larger income being provided by the inhabitants of the city, subscriptions, collections church, and legacies alike show a falling off. Contributions from the working classes alone have increased, which is in itself a certificate of appreciation of the institution. We hope to hear a better record next year, as the careful management revealed by the fact that whilst the number of in-patients has increased by 240, and out-patients by 1,921, the expenditure has only risen by £1,100, should meet with encouragement that anxiety may be removed in future and prosperity be ensured.

**National Hospital for the Paralyzed and Epileptic,** Queen Square, Bloomsbury.—The 34th annual meeting of governors and subscribers was held at this hospital on Thursday, April 27th, Colonel Porter in the chair. Although we understand a large number of notices had been sent out the meeting was very badly attended, only seven governors being present. The necessary business was very quickly dispatched, the chairman remarking he thought so much was seldom got through in so short a time. The usual votes of thanks were cordially given to the board of management, the medical and surgical staff, &c., and Dr. Gibbons, in the course of a few remarks, referred warmly to the excellent administration of the hospital. Speaking from a large experience, he thought it second to none in the country. The patients were excellently well cared for, and the satisfactory results of the surgical operations were quite wonderful. The Chairman, in replying to the vote of thanks to the board of management, said that the members of the board took the deepest interest in the work of the hospital, and their labours were truly "labours of love." At the close of the meeting an announcement was made of the election of pensioners upon the fund for the incurable. From the report we learn that the new wing of the hospital, named after the President, the Duke of Westminster, is proving a very valuable addition to the institution. The board are able to report that the financial conditions have improved, owing to the assistance received from various legacies during the year, but they regret to record a falling off in the income derived from donations. The President has generously increased his subscription from this year to £100, an example which it is to be hoped may be followed by others who are interested in this charity.

